# Antiphospholipid Antibodies Are Major Risk Factors for Non-Thrombotic Cardiac Complications in Systemic Lupus Erythematosus

**DOI:** 10.3390/biomedicines12030530

**Published:** 2024-02-27

**Authors:** Nikolett Nagy, Bernadett Bói, Gábor Papp, Edit Fiák, Eszter Gáspár-Kiss, Bianka Perge, Nikolett Farmasi, Tünde Tarr

**Affiliations:** 1 Division of Clinical Immunology, Institute of Internal Medicine, Faculty of Medicine, University of Debrecen, H-4032 Debrecen, Hungary; nagy.nikolett@med.unideb.hu (N.N.); papp.gabor@med.unideb.hu (G.P.); gaspar.kiss.eszter@med.unideb.hu (E.G.-K.); pergebianka@med.unideb.hu (B.P.); farmasi.nikolett@med.unideb.hu (N.F.); 2 Department of Public Health and Epidemiology, Faculty of Medicine, University of Debrecen, H-4028 Debrecen, Hungary; boi.bernadett97@gmail.com; 3 Department of Cardiology, Faculty of Medicine, University of Debrecen, H-4032 Debrecen, Hungary; dr.fiak.edit@med.unideb.hu

**Keywords:** systemic lupus erythematosus, antiphospholipid antibodies, non-thrombotic cardiac manifestations, aGAPSS

## Abstract

In systemic lupus erythematosus (SLE), cardiovascular complications are among the leading causes of death. Cardiovascular risk in SLE is even higher in the presence of antiphospholipid antibodies or secondary antiphospholipid syndrome (APS). The aim of this retrospective, single-center study was to investigate the occurrence of antiphospholipid antibodies and non-thrombotic cardiac manifestations in 369 SLE patients. We also assessed the clinical and laboratory characteristics of the patients to reveal the risk factors for cardiac manifestations. Patients were divided into two groups based on the presence of antiphospholipid antibodies (APA); 258 (69.9%) patients were APA positive, and 111 (30.1%) patients were APA negative. Mitral and tricuspid insufficiency, aortic stenosis and pulmonary arterial hypertension were more common in APA-positive patients. Anticardiolipin IgG showed the strongest correlation with any non-thrombotic cardiac manifestations. Based on our results, the adjusted global antiphospholipid syndrome score (aGAPSS) above 8.5 is predictive of valvulopathies and ischemic heart disease, while aGAPSS above 9.5 is predictive of cardiomyopathies. The presence of antiphospholipid antibodies may affect the development of cardiac manifestations in SLE. Periodic cardiological and echocardiographic screening of patients without cardiac complaints, as well as regular monitoring of antiphospholipid antibodies, have great importance during the treatment of SLE patients.

## 1. Introduction

Systemic lupus erythematosus (SLE) is a systemic autoimmune disease affecting several organs, including the cardiovascular system. Among the classification criteria of SLE is also pericarditis, which can occur in up to 11–54% of patients [1]. Myocarditis and endocarditis develop less frequently. Libman–Sacks endocarditis is a special form of nonbacterial thrombotic endocarditis that primarily damages the valves of the left side chamber (mitral followed by aortic), but other valves can be also affected. In addition to these, other valve defects, arrhythmias, cardiomyopathies, heart failure, pulmonary arterial hypertension and acute coronary syndrome arising from accelerated atherosclerosis may also occur in SLE [2,3]. These disorders are of exceptional significance because cardiovascular complications are one of the leading causes of death in SLE [4].

SLE often occurs in association with other autoimmune diseases, most frequently with antiphospholipid syndrome (APS). APS is characterized by recurrent arterial and/or venous thrombotic events and a defined group of obstetric complications [5,6]. Antiphospholipid antibodies (APAs), which can be detected in up to 40% of lupus patients, or can be even higher based on their own results, play a crucial role in the development of these disorders [7]. Several antiphospholipid antibodies are known, of which the three most common are the anti-beta2 glycoprotein I antibodies (aß2GPI), the anticardiolipin antibodies (aCL) and the lupus anticoagulant (LA). Based on the research so far, it seems that among the antiphospholipid antibodies, the lupus anticoagulant has the most decisive role in the development of both thrombotic and obstetric complications [5]. However, the greatest risk of thrombosis is the triple antiphospholipid antibody positivity [8,9]. It is known that antiphospholipid antibodies affect the development of cardiac manifestations, but the exact pathomechanism is still not fully understood [10]. It is also known that antiphospholipid antibodies contribute not only to the development of thrombotic events, but also to accelerated atherosclerosis [11]. APS may cause cardiac thrombotic events such as myocardial infarction, but in rare cases, intracardial thrombus formation can also occur. Non-thrombotic clinical manifestations can also develop such as valvulopathies, dilated cardiomyopathy or pulmonary arterial hypertension [11,12]. The association of SLE with APS or antiphospholipid antibody positivity may increase the risk of cardiac manifestations. Several clinical symptoms may develop in both diseases during the disease course. Some of the cardiac manifestations cause clinical symptoms only late; therefore, SLE patients should be screened for cardiac damage even in asymptomatic cases [13].

Patients with definitive APS receive anticoagulant therapy; however, the literature data on the primary prevention of antiphospholipid antibody positives without thrombotic symptoms are divided, as well as on when immunosuppressive treatment is necessary [14,15,16,17]. It is also not yet fully understood which APS patients we can expect to develop recurrent thrombotic events. The Global Antiphospholipid Syndrome Score (GAPSS) is used to estimate the risk of recurrent thrombosis, which takes into account the traditional risk factors such as hypertension and hyperlipidemia, as well as the presence of antiphospholipid antibodies (LA, aCL IgG and/or IgM, aß2GPI IgG and/or IgM and anti-phosphatidylserine/prothrombin complex IgG or IgM). In the case of GAPSS above 10, the risk of developing a thrombotic event is high, but there is no data on whether it is predictive of the development of non-thrombotic APS manifestations [18].

The objective of our study was to assess the occurrence of antiphospholipid antibodies, the manifestations of SLE and APS, and, furthermore, the thrombotic and non-thrombotic cardiac morbidities in a large number of our SLE patients.

## 2. Materials and Methods

### 2.1. Study Population

The Division of Clinical Immunology, Institute of Internal Medicine, Faculty of Medicine, University of Debrecen is one of the largest tertiary referral centers in Hungary for systemic autoimmune diseases. In our retrospective study, we assessed the data of 369 Hungarian patients with SLE, who were diagnosed between 1 January 1977 and 31 December 2018 and followed up regularly at our center, in which at least one transthoracic echocardiography was performed. All patients enrolled in the study have met the 2019 SLE EULAR/ACR Classification Criteria [19]. Patients diagnosed with secondary APS fulfilled both the 2006 Sydney Classification Criteria and the revised ACR/EULAR APS classification criteria, as well [6,20]. This study was approved by the Ethics Committee of our University (protocol number: 4879-2017) and was performed in agreement with the ethical standards of the Declaration of Helsinki.

### 2.2. Clinical and Laboratory Evaluation

All patients were routinely followed up with throughout the studied period, and their medical records contained detailed information on medical history, treatments as well as clinical symptoms, physical conditions and laboratory and other findings of each visit. The following demographic and clinical data were analyzed: age, sex, age at diagnosis, disease duration, organ manifestations of SLE and APS, thrombotic and non-thrombotic cardiac events, hypertension, hyperlipidemia and immunoserological abnormalities during the disease course. Transthoracic echocardiography (TTE) was performed in all patients using a standardized protocol that included M-mode, 2-dimensional (2-D) and Doppler recordings. Valvular lesions were classified by valvular thickness and/or dysfunction (without the presence of vegetations) and pseudo-infective endocarditis. Valvular vegetation was defined as an abnormal localized echodensity with well-defined borders that was either part of or adjacent to valve leaflets, the subvalvular apparatus, or the great vessels. TTE was also used for the assessment of left and right heart function, the presence of segmental disorders in left ventricle kinetics due to coronary artery disease presence, and for the estimation of pressures in the pulmonary vasculature. Cardiomyopathy is defined as the presence of a progressive disorder that impairs the structure and/or function of the muscles in the ventricles of the heart. Data on the history of previous myocardial infarction, episodes of acute heart failure and unstable angina were also collected from patients’ medical records. Hypertension was defined as systolic/diastolic blood pressure ≥ 140/90 mmHg; hyperlipidemia referred to altered levels of blood lipids (triglyceride level ≥ 1.7 mmol/L, total cholesterol level ≥ 5.2 mmol/L, LDL cholesterol > 3.4 mmol/L and/or HDL cholesterol < 1.0 mmol/L). The assessment of chronic organ damage was performed using the SLICC/ACR Damage Index (SDI) to identify chronic organ damage in lupus patients. As we do not routinely check for anti-prothrombin and anti-phosphatidyl-serine antibodies, we used the adjusted aGAPSS score during our studies. Diagnostic laboratory tests, including hemostasis tests for lupus anticoagulant, detection of IgG and IgM type aCL antibodies (positive if >20U/mL), and IgG and IgM type aß2GPI antibodies (positive if >20U/mL), detection of autoantibodies (adsDNA, aSm, aSS-A, aSS-B, ENA, ANA, aRNP), as well as other routine laboratory testing (blood count, ions, liver and kidney function, lipid panel, etc.) were performed in the Department of Laboratory Medicine, Faculty of Medicine, University of Debrecen. The clinical and laboratory data of patients were extracted from medical documentation and records (e-Medsolution and UDMed system) for statistical analyses.

### 2.3. Statistical Analysis

The statistical analysis was performed by using SPSS Statistics for Windows, Version 28.0 (IBM Corporation, Armonk, NY, USA) and GraphPad Prism version 9.5 for Windows (GraphPad Software, San Diego, CA, USA). Kolmogorov–Smirnov and Shapiro–Wilk normality tests were used to determine the distribution of data. In cases of normal distribution, we determined mean ± standard deviation (SD) values and used a two-sample *t*-test for statistical evaluation of the experimental data. In cases of non-normal distribution, median values and interquartile ranges were calculated, and the Mann–Whitney *U* test was used. The chi-squared test and Fisher’s exact test were used to discriminate between patient groups. Univariate and multivariate logistic regression analyses were used to determine the association between APA positivity and cardiac manifestations. The rank biserial correlation was used to assess the relationship between a dichotomous categorical variable and an ordinal variable. We determined the optimal cut-off value for aGAPSS with ROC curve analysis. We used two-sided statistical tests, where the *p* value represented a significance rate of <0.05.

## 3. Results

### 3.1. Main Analyses

The population of our retrospective study consisted of 369 Hungarian patients with SLE (336 women and 33 men; sex ratio = 10.2 to 1). Their age at the time of SLE diagnosis was 32.3 ± 11.6 years and they were followed up with regularly at our Autoimmune Center for 17.3 ± 10.1 years.

Patients were divided into two groups based on APA positivity. Of the 369 patients included in our study, 258 patients (69.9%) were in the APA-positive group, while 111 patients (30.1%) were in the APA-negative group. All patients were white adults and the demographic characteristics of the patients’ groups did not differ significantly (Table 1).

### 3.2. Antiphospholipid Antibodies and Other Laboratory Parameters in APA+ and APA− Patients

**Figure 1:**
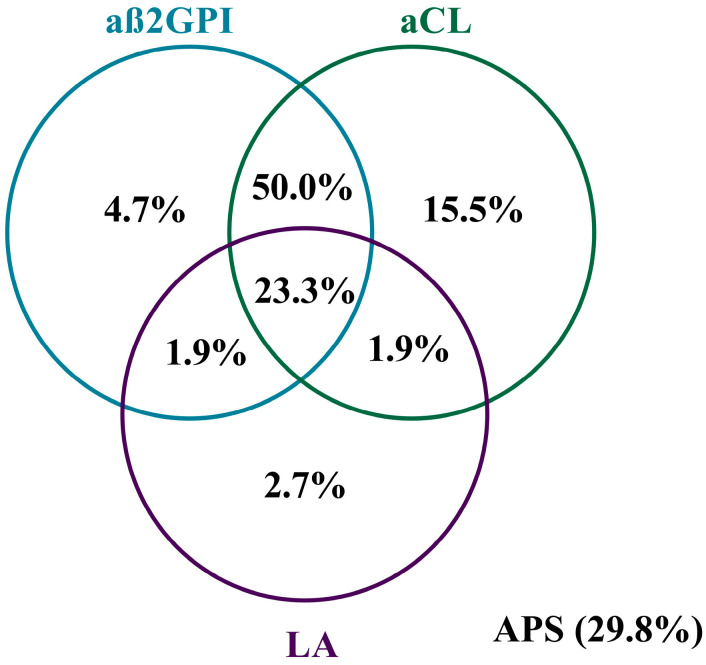
Frequency of antibody positivity in the APA+ group. The Venn diagram shows the frequency of single, double and triple antibody positivity for aß2GPI, aCL and LA in 258 patients. The frequency of APS is also shown. Abbreviations: APS, antiphospholipid syndrome; LA, lupus anticoagulant; aCL, anticardiolipin antibody; aß2GPI, anti-β2-glycoprotein-1 antibody.

Figure 1 demonstrates the prevalence of antiphospholipid antibodies and their association with each other. The aß2GPI + aCL positivity occurred most frequently (50.0%), followed by aß2GPI + aCL + LA positivity (23.3%). Isolated aCL positivity was found in 15.5%, isolated aß2GPI in 4.7% and isolated LA positivity in 2.7% of the cases. The aß2GPI + LA and aCL + LA combinations were detected in 1.9% of the cases. In total, 29.8% of all patients were diagnosed with APS.

Several other laboratory abnormalities were found to be more frequent in the APA+ group compared to the APA− group. We observed significant difference in the following parameters: thrombocytopenia (43.0% vs. 30.6%), anemia (82.2% vs. 64.9%), anti-dsDNA (95.7% vs. 82.0%), anti-Sm (41.1% vs. 22.5%), anti-RNP (36.0% vs. 20.7%), anti-SS-A (70.2% vs. 59.5%), ANCA (14.0% vs. 1.8%) and cryoglobulinemia (5.4% vs. 0.9%), as shown in Table 2.

### 3.3. Clinical Manifestations and Steroid Treatment in APA+ and APA− Patients

Regarding the organ manifestation and laboratory abnormalities of SLE, the occurrence of alopecia (27.9% vs. 14.4%), central nervous system symptoms (28.3% vs. 12.6%), peripheral nervous system symptoms (12.4% vs. 5.4%) and psychiatric disorders (23.3% vs. 12.6%) were significantly more common in the APA-positive group. However, the prevalence of subacute cutaneous lupus (SCLE) was significantly lower in the APA-positive group compared to the APA-negative group (7.0% vs. 20.7%). Furthermore, the SLICC/ACR SDI value [1 (0–2) point vs. 1 (0–1) point] and also the median cumulative steroid dose [197,10 (8760–35,030) mg/kg vs. 11,680 (4380–27,740) mg/kg] were significantly higher in the APA+ group compared to the APA− group (Table 3).

### 3.4. Cardiac Manifestations in APA+ and APA− Patients

We detected non-thrombotic cardiac manifestations in 171 (43.6%) patients among all the examined patients. Valvulopathies were the most common cardiac abnormality. In the group of all patients, mitral insufficiency (MI) was diagnosed in 29.5% of patients, while tricuspid insufficiency (TI) was found in 26.8% of patients. The prevalence of ischemic heart disease (IHD) was 7.9%, aortic insufficiency (AI) was 6.2% and cardiomyopathy was 5.4%. The proportion of patients who had an acute myocardial infarction (AMI) was 3.5%.

All cardiac manifestations were more common in the APA+ group compared to the APA− group, but a significant difference was only in the case of tricuspid (31.4% vs. 18.0%) and mitral insufficiency (33.7% vs. 21.6%). Pulmonary hypertension and Libman–Sacks endocarditis occurred only in the APA+ group (Table 4). We performed univariate and multivariate logistic regression analyses to determine the association between APA positivity and cardiac manifestations using variables with *p* < 0.05 in the chi-squared test. Univariate analysis revealed that APA positivity was significantly associated with MI (odds ratio (OR) = 1.84, 95% confidence interval (CI) 1.10–3.10, *p* = 0.021) and TI (OR = 2.08, 95% CI 1.20–3.61, *p* = 0.009) risk. The results were not changed after adjustment for gender, age at SLE diagnosis, duration of SLE, hyperlipidemia and hypertonia (MI OR = 2.17, 95% CI 1.24–3.80, *p* = 0.007; TI OR = 2.46, 95% CI 1.37–4.41, *p* = 0.003).

### 3.5. Differences between Patients with and without Cardiac Manifestations

As a next step, we compared the patients with cardiac manifestations with the ones without cardiac diseases. We found that anti-CL IgG (81.4% vs. 16.3%) and anti-CL IgM (78.3% vs. 14.7%), as well as aß2GPI IgG (74.4% vs. 17.1%) and aß2GPI IgM (68.2% vs. 15.5%), were significantly more prevalent in the patients who developed some kind of cardiac manifestation. Double and triple APA positivity did not differ between patient groups.

The prevalence of hypertonia (63.2% vs. 22.2%) and hyperlipidemia (49.7% vs. 16.7%) were significantly higher in patients with cardiac manifestations, and the frequency of stroke was also significantly higher in this group (10.5% vs. 3.5%) (Table 5).

Since valvulopathies were the most common cardiac abnormalities, we also compared patients with and without valvulopathy. In the group of patients with valvulopathies, we observed a higher prevalence of aCL IgG (80.9% vs. 23.1%), aCL IgM (77.4% vs. 21.7%), aß2GPI IgG (73% vs. 23.8%) and aß2GPI IgM (66.1% vs. 22.4%) positivity. Additionally, we found a higher prevalence of hypertension (50.3% vs. 27.3%), hyperlipidemia (50.3% vs. 19%), ischemic heart disease (13.1% vs. 4.2%) and cardiomyopathy (9.8% vs. 2.8%) in this patient group (Table 6).

### 3.6. Characteristics of the APA+ Patients

In the following, we focused on the APA-positive patients (*n* = 258). Among them, lupus anticoagulant (17.8%) was the least frequently detected, while the occurrence of aCL IgM was 44.6%, aCL IgG was 48.5%, aß2GPI IgM was 41.5% and aß2GPI IgG was 45.4%. A positive aGAPSS was detected in 148 patients (57.4%). We did not find a significant difference in the prevalence of cardiac manifestations when comparing the single APA-positive group with either the double APA-positive or the triple APA-positive group. Deep venous thrombosis as well as definitive APS were diagnosed significantly more frequently in triple APA-positive patients (Table 7).

**Figure 2:**
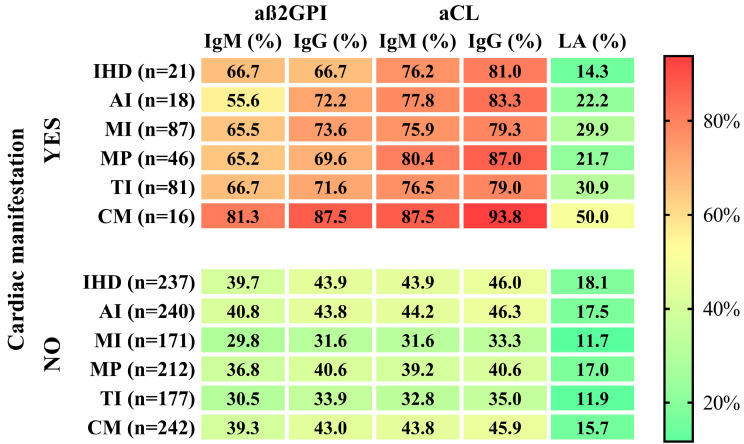
Heat map shows the frequency of aß2GPI IgM/IgG, aCL IgM/IgG and LA in the presence (YES) and absence (NO) of cardiac manifestation. Abbreviations: IHD, ischemic heart disease; AI, aortic insufficiency; MI, mitral insufficiency; MP, mitral prolapse; TI, tricuspidal insufficiency; CM, cardiomyopathy; aß2GPI, anti-β2-glycoprotein-1 antibody; aCL, anticardiolipin antibody; IgM, immunoglobulin M; IgG immunoglobulin G; LA, lupus anticoagulant.

In the further analyses for the APA+ group, the cardiac manifestations that at least 10 patients had were included in the statistical analyses suitable for examining the correlation between individual variables. We examined the associations between the isotypes of each antiphospholipid antibody and ischemic heart disease, mitral prolapse, mitral insufficiency, tricuspid insufficiency, aortic insufficiency and cardiomyopathy. Figure 2 shows the occurrence of specific antibodies in the presence or absence of various heart diseases. Anticardiolipin IgG positivity was the most common, while lupus anticoagulant positivity was the least common in the case of all examined cardiac manifestations.

### 3.7. The Associations between the APAs and aGAPSS Values and the Specific Cardiac Diseases

**Figure 3:**
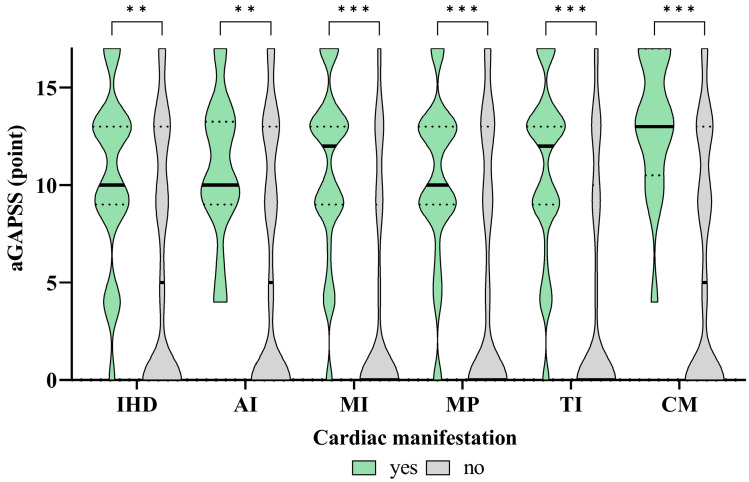
Level of aGAPSS among patients with different cardiac manifestations. On the violin plot black solid line shows the median aGAPSS, and IQR represented by black dotted line. *p*-values were calculated by Mann–Whitney U test, reported as ** *p* < 0.01, *** *p* < 0.001. Abbreviations: IHD, ischemic heart disease; AI, aortic insufficiency; MI, mitral insufficiency; MP, mitral prolapse; TI, tricuspidal insufficiency; CM, cardiomyopathy; aGAPSS, Adjusted Global Antiphospholipid Syndrome Score.

**Figure 4:**
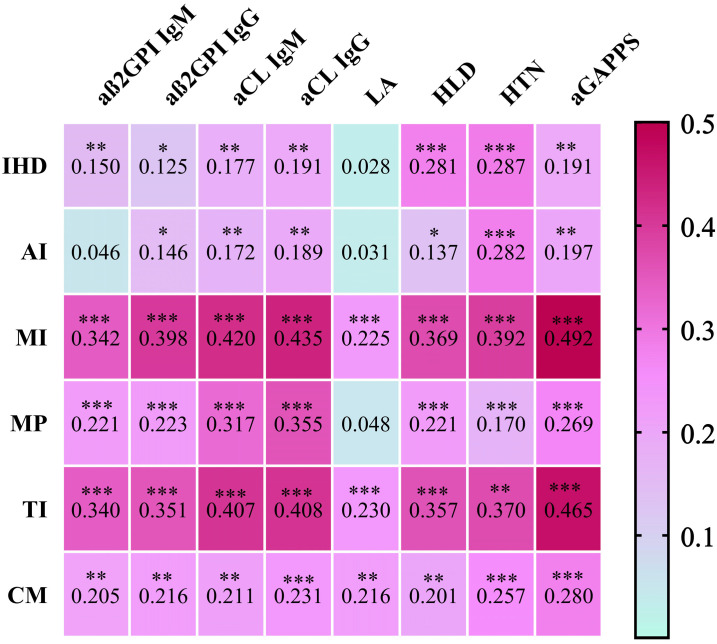
Correlation matrix on the relationship between cardiac manifestations and aGAPPS. The relationship between aGAPPS variables and cardiac manifestations is shown by the Cramer’s V value from Pearson’s chi-squared test, while the relationship between aGAPSS and cardiac manifestations is shown by the rrb coefficient from rank-biserial correlation. * *p* < 0.05, ** *p* < 0.01, *** *p* < 0.001. Abbreviations: IHD, ischemic heart disease; AI, aortic insufficiency; MI, mitral insufficiency; MP, mitral prolapse; TI, tricuspidal insufficiency; CM, cardiomyopathy; aß2GPI, anti-β2-glycoprotein-1 antibody; aCL, anticardiolipin antibody; IgM, immunoglobulin M; IgG, immunoglobulin G; LA, lupus anticoagulant; HLD, hyperlipidemia; HTN, hypertonia; aGAPSS, Adjusted Global Antiphospholipid Syndrome Score.

The associations between the aGAPSS values and the specific cardiac diseases are shown in Figure 3 and Figure 4.

Regarding IHD, the IgM and IgG isotypes of aß2GPI and aCL were significantly more prevalent in this condition; however, the effect size is small. There was no significant difference in the case of lupus anticoagulant, while the aGAPSS values show a negligible but statistically significant correlation with IHD. Regarding AI, the aß2GPI IgG and aCL IgM/IgG isotypes are significantly more prevalent in this disorder, but the effect size is small. There was no significant difference in the case of aß2GPI IgM and LA, while the aGAPSS values show a negligible but statistically significant correlation with AI. In TI and MI patients, aß2GPI, aCL IgM and IgG isotypes, as well as LA, were significantly more common, and the effect size is medium–large in the case of these antibodies (apart from LA, in which it is small). The aGAPSS values show a medium–large significant correlation with TI and MI. Regarding MP, the aß2GPI and aCL IgM and IgG isotypes were significantly more common in this disorder; the effect size for aß2GPI immunoglobulins is small, while for aCL immunoglobulins it is medium–large. There was no significant difference in the case of LA. The aGAPSS shows a weak, but significant correlation with MP. As for CM patients, the aß2GPI, aCL IgM and IgG isotypes, as well as LA, were significantly more prevalent; however, the effect size is small. The aGAPSS values show a weak, but significant correlation with CM. Out of all antibodies, the largest effect size was found in aCL IgG in the case of all cardiac manifestations.

Based on our results, we tried to find an aGAPSS score in which a non-thrombotic cardiac manifestation is more likely to occur (Table 8). In the case of cardiomyopathy, the aGAPSS value above 9.5 points, while in the case of the other investigated cardiac deviations, the aGAPSS value above 8.5 points seems to be predictive of the development of the given cardiac manifestation.

## 4. Discussion

Cardiac manifestations are common in both SLE and antiphospholipid syndrome; nevertheless, only pericarditis is included in the classification criteria of SLE. Formerly, the thrombotic arterial event was the only cardiac manifestation in the criteria for APS, but the new ACR/EULAR classification criteria for APS include cardiac valve thickening and vegetation as well [20]. In addition to these, many cardiac abnormalities can occur in both diseases [1,12]. Among the non-criteria symptoms, the most common in both diseases is the development of various valvulopathies [1,12]. With the spread of echocardiography, the diagnosis of valvular disorders associated with SLE and APS has become more accurate and earlier. The frequency of occurrence varies by cohort, but it affects approximately 40% of patients, as we also found (41.5%). The pathomechanism of valvulopathies is not yet fully understood. Based on the histological examination of the autopsy samples, mononuclear cell infiltration, endothelial cell proliferation, platelet activation and mycrothrombus formation can be observed [10]. It has been suggested that APA positivity increases the risk of valve thrombi formation, which may explain the association of APAs with valvular lesions [21]. Moreover, autopsy data on APA-positive patients with dilated cardiomyopathy have shown microthrombotic occlusion of myocardial arterioles, which potentially contributed to the dysfunction of the heart [13]. Presumably, similar microthrombotic occlusions in the arterial supply of valves may also be a contributing factor to the valvular dysfunction in APA-positive patients. Possibly, APAs may also cause subendocardial inflammation through an interaction with antigens on valve surfaces. The mechanisms of thrombosis and inflammation subsequently lead to fibrosis and valve deformation [12].

Previous studies confirmed that antiphospholipid antibodies may be associated with cardiovascular diseases in the general population as well. The presence of APAs impacts accelerated atherosclerosis and may lead to cardiovascular complications through multiple mechanisms, such as inhibition of the attachment of the plaque stabilizing and antithrombotic plasma protein, annexin-V to the endothelium, as well as the increase in lipid peroxidation, or the identification of the oxidated phospholipids [22,23].

The purpose of our present study was to assess the prevalence of non-thrombotic cardiac manifestations in SLE patients and evaluate the connection between these disorders and the presence of specific antiphospholipid antibodies. We were also interested in whether the aGAPPS score is predictive of the development of non-thrombotic cardiac manifestations.

All cardiac diseases were more common in the APA-positive group of our patient population, but a significant difference was found only in the case of mitral and tricuspid insufficiency. Univariate and multivariate logistic regression analyses also confirmed the association between APA positivity and these heart valve defects. Similar results were previously described by several working groups, while others found no correlation [24,25,26,27]. Multiple studies examined the relationship between the isotype or titer of antiphospholipid antibodies and valvular diseases [28,29]; however, data in the literature are controversial. Pons and colleagues found that with the presence of lupus anticoagulant or double antiphospholipid antibody positivity, valvular disease has a higher chance of developing. They also found that valvulopathies are more common in arterial hypertension and arterial thrombosis at the diagnosis of APS, as well as in livedo reticularis. They found no difference between the primary APS and secondary SLE-associated APS patients [30]. Djokovic and colleagues analyzed a total of 374 patients, out of whom 260 patients were diagnosed with primary APS and 114 patients had SLE-associated secondary APS. Cardiac manifestations showed a significantly higher prevalence in SLE-associated secondary APS patients according to their results, and they found valvulopathies to be the most common disease, which is in accordance with our results. Valvulopathies showed an association with anticardiolipin IgG and acute heart failure, whereas myocardial infarction was associated with lupus anticoagulants [31].

Analyzing our own patient population, we found that stroke was more common in patients who showed any form of cardiac manifestation. Another working group also found the occurrence of stroke to be more frequent, primarily among SLE patients with valvulopathy [32]. Traditional cardiovascular risk factors such as hypertension and hyperlipidemia were more common among patients with cardiac manifestations, which we know contribute significantly to the development of cardiovascular diseases in addition to disease-specific risk factors.

Our results showed that anticardiolipin IgG and IgM, as well as anti-ß2GPI IgG and IgM, were more common in patients with valvular diseases. Lupus anticoagulant did not affect the occurrence of cardiac manifestations, including valvulopathies. We did not find a correlation between double or triple antiphospholipid antibody positivity and any specific cardiac diseases. Definitive APS was diagnosed significantly more frequently in patients with triple APA positivity; however, this did not have an impact on the development of thrombotic and non-thrombotic cardiac events. Based on our data, we found the strongest association between the presence of anticardiolipin IgG antibodies and non-thrombotic cardiac manifestations. Based on the results of a recently published meta-analysis, patients with anticardiolipin IgG antibodies can benefit the most from echocardiography screening tests [10]. At the same time, the incidence of ischemic heart disease and cardiomyopathy was significantly higher in patients with valvulopathy, which draws attention to the fact that non-thrombotic cardiac manifestations can occur not only independently but also in combination with each other.

In our study population, the incidence of ischemic heart disease, aortic insufficiency and cardiomyopathy was less than 10%, while the proportion of patients with acute myocardial infarction was 3.5%. International data show that pulmonary arterial hypertension develops in less than 4% of SLE patients, which is in accordance with our own observations, as PAH occurred in 1.08% of our patient population, noting that this only occurred in antiphospholipid antibody-positive patients [33].

We evaluated the possible correlations between the GAPSS used to estimate the risk of recurrent thrombosis and the development of non-thrombotic cardiac manifestations. Regarding this question, only a few datasets are available in the literature. Previous studies primarily targeted the association between the aGAPSS and myocardial infarction. A high aGAPSS score may be helpful in assessing the risk of myocardial infarction according to their results [34]. Song et al. investigated the aGAPPS score in primary APS patients in relation to the estimation of ischemic stroke. They found that an aGAPSS above 10 significantly increased the risk of stroke [35]. Furthermore, a Spanish working group examined whether the aGAPSS score is predictive of the development of obstetrical complications in patients with antiphospholipid antibody positivity. They found that the aGAPPS score cannot be used for risk estimation in this population, since in addition to antiphospholipid antibodies, several other pregnancy-specific factors influence pregnancy outcome [36]. In our study, we found a positive aGAPSS in 148 patients (57.4%) within the APA+ patient group. IHD and AI showed a negligible, CM a weak, while TI and MI showed a medium correlation with the aGAPSS values. Based on our results, in the case of cardiomyopathy, the aGAPSS value above 9.5 points, while in the case of the other investigated cardiac deviations, the aGAPSS value above 8.5 points seems to be predictive of the development of the given cardiac manifestation. Similar results and studies were not found in the literature.

In conclusion, our study confirmed that the presence of antiphospholipid antibodies not only increases the occurrence of thrombotic cardiac manifestations, but of valvular diseases also in the Hungarian SLE population. According to our results, the strongest association can be observed between non-thrombotic cardiac manifestations and anticardiolipin IgG antibodies. Regular screening of SLE patients with echocardiography is strongly recommended especially in case of patients presenting anticardiolipin IgG antibodies or having an aGAPSS above 8.5. When detecting valvulopathies, we should also consider the development of cardiomyopathy and ischemic heart disease more often.

## Figures and Tables

**Table 1:** Demographic characteristics of SLE patients in APA− and APA+ groups.

	SLE Cohort (*n* = 369)	APA− (*n* = 111)	APA+ (*n* = 258)	*p*-Value
Sex (women, %)	336 (91.1)	100 (90.1)	236 (91.5)	0.669
Age, years	49.7 ± 13.4	48.8 ± 14.3	50.1 ± 13.1	0.401
Age onset SLE, years	32.3 ± 11.6	32.4 ± 11.9	32.3 ± 11.5	0.998
Duration of SLE, years	17.3 ± 10.1	16.4 ± 11.8	17.1 ± 9.2	0.310

Values are presented as number (%), and mean ± SD, *p*-values were calculated by Pearson’s chi-squared test and Student’s *t*-test for two samples. Abbreviations: *n*, number of patients; APA−, antiphospholipid antibody and antiphospholipid syndrome negative; APA+, antiphospholipid antibody or antiphospholipid syndrome positive.

**Table 2:** Positive laboratory findings in APA− and APA+ groups.

	SLE Cohort (*n* = 369)	APA− (*n* = 111)	APA+ (*n* = 258)	*p*-Value
Thrombocytopenia	145 (39.3)	34 (30.6)	111 (43.0)	**0.025**
Leukopaenia	269 (72.9)	78 (70.3)	191 (74.0)	0.456
Anaemia	284 (77.0)	72 (64.9)	212 (82.2)	**<0.001**
Anti-dsDNA	338 (91.6)	91 (82.0)	247 (95.7)	**<0.001**
Anti-Sm	131 (35.5)	25 (22.5)	106 (41.1)	**0.001**
Anti-RNP	116 (31.4)	23 (20.7)	93 (36.0)	**0.004**
Anti-SS-A (Ro)	247 (66.9)	66 (59.5)	181 (70.2)	**0.045**
Anti-SS-B (La)	168 (45.5)	46 (41.4)	122 (47.3)	0.301
ANCA	38 (10.3)	2 (1.8)	36 (14.0)	**<0.001**
Cryoglobulin	15 (4.1)	1 (0.9)	14 (5.4)	**0.046**
Coombs test positivity	41 (11.1)	10 (9.0)	31 (12.0)	0.399

Values are presented as number (%), *p*-values were calculated by Pearson’s chi-squared test or Fisher’s exact test. Bold indicates statistically significant (*p* < 0.05) result. Abbreviations: *n*, number of patients; APA−, antiphospholipid antibody and antiphospholipid syndrome negative; APA+, antiphospholipid antibody or antiphospholipid syndrome positive; anti-dsDNA, anti-double-stranded DNA; anti-Sm, anti-Smith antibody; anti-RNP, anti-RNP, anti-ribonucleoprotein antibody; anti-SS-A, anti-Sjögren’s-syndrome-related antigen A autoantibody; anti-SS-B, anti-Sjögren’s-syndrome-related antigen B autoantibody; ANCA, antineutrophil cytoplasmic antibody.

**Table 3:** Clinical manifestations of SLE in APA− and APA+ groups.

	SLE Cohort (*n* = 369)	APA− (*n* = 111)	APA+ (*n* = 258)	*p*-Value
Acute skin lesions	151 (40.9)	41 (36.9)	110 (42.6)	0.307
DLE	52 (14.1)	17 (15.3)	35 (13.6)	0.658
SCLE	41 (11.1)	23 (20.7)	18 (7.0)	**<0.001**
Alopecia	88 (23.8)	16 (14.4)	72 (27.9)	**0.005**
Photosensitivity	101 (27.4)	31 (27.9)	70 (27.1)	0.875
Mucous ulcer	35 (9.5)	15 (13.5)	20 (7.8)	0.083
Pleuritis	94 (25.5)	27 (24.3)	67 (26.0)	0.739
Pericarditis	61 (16.5)	19 (17.1)	42 (16.3)	0.842
CNS manifestations	87 (23.6)	14 (12.6)	73 (28.3)	**0.001**
PNS manifestations	38 (10.3)	6 (5.4)	32 (12.4)	**0.043**
Psychiatric manifestations	74 (20.1)	14 (12.6)	60 (23.3)	**0.019**
LN	114 (30.9)	30 (27.0)	84 (32.6)	0.292
Polyarthritis	314 (85.1)	92 (82.9)	222 (86.0)	0.434
Cumulative dose of steroid, mg/kg	17,520 (7300–32,120)	11,680 (4380–27,740)	19,710 (8760–35,030)	**0.003**
SDI, points	1 (0-1)	1 (0-1)	1 (0-2)	**0.001**

Values are presented as number (%) and median with IQR, *p*-values were calculated by Pearson’s chi-squared and Mann–Whitney U test. Bold indicates statistically significant (*p* < 0.05) result. Abbreviations: *n*, number of patients; APA−, antiphospholipid antibody and antiphospholipid syndrome negative; APA+, antiphospholipid antibody or antiphospholipid syndrome positive. DLE, discoid lupus erythematosus; SCLE, subacute cutaneous lupus; CNS, central nervous system; PNS, peripheral nervous system; LN, lupus nephritis; SDI, SLICC/ACR Damage Index.

**Table 4:** Cardiac manifestations in the APA− and APA+ groups.

	SLE Cohort (*n* = 369)	APA− (*n* = 111)	APA+ (*n* = 258)	*p*-Value
Ischemic heart disease	29 (7.9)	8 (7.2)	21 (8.1)	0.760
Valvulopathy	153 (41.5)	38 (34.2)	115 (44.6)	0.064
Aortic insufficiency	24 (6.5)	6 (5.4)	18 (7.0)	0.575
Aortic stenosis	4 (1.1)	0	4 (1.6)	0.320
Pulmonary insufficiency	3 (0.8)	0	3 (1.2)	0.557
Mitral insufficiency	111 (30.1)	24 (21.6)	87 (33.7)	**0.020**
Mitral prolapse	61 (16.5)	15 (13.5)	46 (17.8)	0.306
Tricuspid insufficiency	101 (27.4)	20 (18.0)	81 (31.4)	**0.008**
Cardiomyopathy	21 (5.7)	5 (4.5)	16 (6.2)	0.519
Pulmonary hypertension	5 (1.4)	0	5 (1.9)	0.328
Libman–Sacks endocarditis	2 (0.5)	0	2 (0.8)	1.000
Hyperlipidemia	118 (32.0)	35 (31.5)	83 (32.2)	0.904
Hypertonia	152 (41.2)	52 (46.8)	100 (38.8)	0.148

Values are presented as number (%), *p*-values were calculated by Pearson’s chi-squared test or Fisher’s exact test. Bold indicates statistically significant (*p* < 0.05) result. Abbreviations: *n*, number of patients; APA−, antiphospholipid antibody and antiphospholipid syndrome negative; APA+, antiphospholipid antibody or antiphospholipid syndrome positive.

**Table 5:** Demographic characteristics, autoantibodies, cardiovascular risk factors, manifestations of APS in SLE patients with and without cardiac manifestations.

	SLE Cohort (*n* = 369)	Without Cardiac Manifestations (*n* = 198; 53.7%)	With Cardiac Manifestations (*n* = 171; 43.6%)	*p*-Value
Demographic				
Gender (female)	336 (91.1)	180 (90.9)	156 (91.2)	0.915
Age at SLE onset (years)	32.3 ± 11.6	31.9 ± 11.7	32.8 ± 11.5	0.444
Duration of SLE (years)	17.3 ± 10.1	16.4 ± 9.5	18.4 ± 10.7	0.068
Autoantibodies				
LA	77 (20.9)	39 (19.7)	38 (22.2)	0.552
aß2GPI	206 (55.8)	103 (52)	103 (60.2)	0.113
aß2GPI IgM	108 (41.9)	20 (15.5)	88 (68.2)	**<0.001**
aß2GPI IgG	118 (45.7)	22 (17.1)	96 (74.4)	**<0.001**
aCL	234 (63.4)	119 (60.1)	115 (67.3)	0.155
aCL IgM	120 (46.5)	19 (14.7)	101 (78.3)	**<0.001**
aCL IgG	126 (48.8)	21 (16.3)	105 (81.4)	**<0.001**
Single antibody positivity	59 (16)	28 (14.1)	31 (18.1)	0.297
LA	7 (1.9)	3 (1.5)	4 (2.3)	0.709
aß2GPI	12 (3.3)	5 (2.5)	7 (4.1)	0.397
aCL	40 (10.8)	20 (10.1)	20 (11.7)	0.623
Double antibody positivity	139 (37.7)	70 (35.4)	69 (40.4)	0.323
LA + aß2GPI	5 (1.4)	2 (1.0)	3 (1.8)	0.666
LA + aCL	5 (1.4)	3 (1.5)	2 (1.2)	1.000
aß2GPI + aCL	129 (35.0)	65 (32.8)	64 (37.4)	0.356
Triple antibody positivity	60 (16.3)	31 (15.7)	29 (17)	0.735
Cardiovascular risk factors				
Hyperlipidemia	118 (32.0)	33 (16.7)	85 (49.7)	**<0.001**
Hypertonia	152 (41.2)	44 (22.2)	108 (63.2)	**<0.001**
APS	77 (20.9)	36 (18.2)	41 (24.0)	0.172
Deep vein thrombosis	68 (18.4)	39 (19.7)	29 (17.0)	0.499
Acute myocardial infarction	13 (3.5)	5 (2.5)	8 (4.7)	0.263
Pulmonary embolism	13 (3.5)	7 (3.5)	6 (3.5)	0.989
Stroke	25 (6.8)	7 (3.5)	18 (10.5)	**0.008**
Spontaneous abortion	49 (13.3)	21 (10.6)	28 (16.4)	0.103
Livedo reticularis	25 (6.8)	11 (5.6)	14 (8.2)	0.316
Thrombocytopenia	145 (39.3)	70 (35.4)	75 (43.9)	0.095

Values are presented as number (%) and mean ± SD, *p*-values were calculated by Pearson’s chi-squared test and Student’s *t*-test for two samples. Bold indicates statistically significant (*p* < 0.05) result. Abbreviations: *n*, number of patients; LA, lupus anticoagulant; aß2GPI, anti-β2-glycoprotein-1 antibody; aCL, anticardiolipin antibody; IgM, immunoglobulin M; IgG, immunoglobulin G; APS, antiphospholipid syndrome.

**Table 6:** Demographic characteristics, autoantibodies, cardiovascular risk factors, manifestations of APS with and without valvulopathy in patients with SLE.

	SLE Cohort (*n* = 369)	Without Valvulopathy (*n* = 216; 58.5%)	With Valvulopathy (*n* = 153; 41.5%)	*p*-Value
Demographic				
Gender (female)	336 (91.1)	195 (90.3)	141 (92.2)	0.533
Age at SLE onset (years)	32.3 ± 11.6	32.2 ± 11.9	32.5 ± 11.2	0.831
Duration of SLE (years)	17.3 ± 10.1	16.9 ± 9.8	17.9 ± 10.5	0.377
Autoantibodies				
LA	77 (20.9)	44 (20.4)	33 (21.6)	0.780
aß2GPI	206 (55.8)	116 (53.7)	90 (58.8)	0.329
aß2GPI IgM	108 (41.9)	32 (22.4)	76 (66.1)	**<0.001**
aß2GPI IgG	118 (45.7)	34 (23.8)	84 (73.0)	**<0.001**
aCL	234 (63.4)	133 (61.6)	101 (66.0)	0.383
aCL IgM	120 (46.5)	31 (21.7)	89 (77.4)	**<0.001**
aCL IgG	126 (48.8)	33 (23.1)	93 (80.9)	**<0.001**
Single antibody positivity	59 (16.0)	28 (13.0)	31 (20.3)	0.059
LA	7 (1.9)	3 (1.4)	4 (2.6)	0.455
aß2GPI	12 (3.3)	5 (2.3)	7 (4.6)	0.247
aCL	40 (10.8)	20 (9.3)	20 (13.1)	0.246
Double antibody positivity	139 (37.7)	80 (21.7)	59 (16.0)	0.766
LA + aß2GPI	5 (1.4)	2 (0.9)	3 (2.0)	0.653
LA + aCL	5 (1.4)	4 (1.9)	1 (0.7)	0.408
aß2GPI + aCL	129 (35.0)	74 (34.3)	55 (35.9)	0.738
Triple antibody positivity	60 (16.3)	35 (16.2)	25 (16.3)	0.972
Anti-dsDNA	338 (91.6)	199 (92.1)	139 (90.8)	0.662
Anti-Sm	131 (35.5)	77 (35.6)	54 (35.3)	0.944
Anti-RNP	116 (31.4)	64 (29.6)	52 (34.0)	0.374
Anti-SS-A (Ro)	247 (66.9)	145 (67.1)	102 (66.7)	0.926
Anti-SS-B (La)	168 (45.5)	98 (45.4)	70 (45.8)	0.942
Cardiac manifestations				
Ischemic heart disease	29 (7.9)	9 (4.2)	20 (13.1)	**0.002**
Cardiomyopathy	21 (5.7)	6 (2.8)	15 (9.8)	**0.004**
Carotid stenosis	5 (1.4)	1 (0.5)	4 (2.6)	0.165
Pulmonary hypertension	5 (1.4)	2 (0.9)	3 (2.0)	0.653
Libman–Sacks endocarditis	2 (0.5)	1 (0.5)	1 (0.7)	1.000
Cardiovascular risk factors				
Hyperlipidemia	118 (32.0)	41 (19.0)	77 (50.3)	**<0.001**
Hypertonia	152 (41.2)	59 (27.3)	93 (60.8)	**<0.001**
APS	77 (20.9)	43 (19.9)	34 (22.2)	0.590
Deep vein thrombosis	68 (18.4)	44 (20.4)	24 (15.7)	0.253
Acute myocardial infarction	13 (3.5)	7 (3.2)	6 (3.9)	0.727
Pulmonary embolism	13 (3.5)	8 (3.7)	5 (3.3)	0.823
Stroke	25 (6.8)	10 (4.6)	15 (9.8)	0.051
Spontaneous abortion	49 (13.3)	24 (11.1)	25 (16.3)	0.145
Livedo reticularis	25 (6.8)	12 (5.6)	13 (8.5)	0.268
Thrombocytopenia	145 (39.3)	80 (37.0)	65 (42.5)	0.291

Values are presented as number (%) and mean ± SD, *p*-values were calculated by Pearson’s chi-squared test and Student’s *t*-test for two samples. Bold indicates statistically significant (*p* < 0.05) result. Abbreviations: *n*, number of patients; LA, lupus anticoagulant; aß2GPI, anti-β2-glycoprotein-1 antibody; aCL, anticardiolipin antibody; IgM, immunoglobulin M; IgG, immunoglobulin G; anti-dsDNA, anti-double-stranded DNA; anti-Sm, anti-Smith antibody; anti-RNP, anti-RNP, anti-ribonucleoprotein antibody; anti-SS-A, anti-Sjögren’s-syndrome-related antigen A autoantibody; anti-SS-B, anti-Sjögren’s-syndrome-related antigen B autoantibody; APS, antiphospholipid syndrome.

**Table 7:** Demographic characteristics, autoantibodies, cardiovascular risk factors, manifestations of APA+ patients with single, double or triple APA positivity.

	Single AB Positivity (*n* = 59; 22.9%)	Double AB Positivity (*n* = 139; 53.9%)	*p*-Value Double vs. Single Pos.	Triple AB Positivity (*n* = 60; 23.3%)	*p*-Value Triple vs. Single Pos.
Demographic					
Gender (female)	57 (96.6)	127 (91.4)	0.188	52 (86.7)	0.095
Age at SLE onset (years)	34 ± 12.0	31.8 ± 11.4	0.215	32.1 ± 11.3	0.371
Duration of SLE (years)	17.7 ± 10.5	18.2 ± 8.2	0.722	16.7 ± 10.2	0.606
Cardiac manifestations					
Ischemic heart disease	7 (11.9)	11 (7.9)	0.376	3 (5.0)	0.204
Valvulopathy	31 (52.5)	59 (42.4)	0.192	25 (41.7)	0.235
Aortic insufficiency	4 (6.8)	10 (7.2)	1.000	4 (6.7)	1.000
Aorta stenosis	0 (0)	4 (2.9)	0.320	0 (0)	n.c.
Tricuspid insufficiency	22 (37.3)	37 (26.6)	0.133	22 (36.7)	0.944
Mitral insufficiency	23 (39.0)	43 (30.9)	0.272	21 (35.0)	0.653
Pulmonary insufficiency	1 (1.7)	2 (1.4)	1.000	0 (0)	0.496
Mitral prolapse	13 (22.0)	24 (17.3)	0.431	9 (15.0)	0.323
Cardiomyopathy	1 (1.7)	9 (6.5)	0.287	6 (10.0)	0.114
Carotid stenosis	1 (1.7)	2 (1.4)	1.000	2 (3.3)	1.000
Pulmonary hypertension	1 (1.7)	1 (0.7)	0.508	3 (5.0)	0.619
Libman–Sacks endocarditis	0 (0)	2 (1.4)	1.000	0 (0)	n.c.
Cardiovascular risk factors					
Hyperlipidemia	18 (30.5)	46 (33.1)	0.722	19 (31.7)	0.891
Hypertonia	19 (32.2)	53 (38.1)	0.428	28 (46.7)	0.107
APS	9 (15.3)	31 (22.3)	0.259	37 (61.7)	**<0.001**
Deep vein thrombosis	4 (6.8)	22 (15.8)	0.085	30 (50.0)	**<0.001**
Acute myocardial infarction	3 (5.1)	4 (2.9)	0.427	4 (6.7)	1.000
Pulmonary embolism	1 (1.7)	1 (0.7)	0.508	6 (10.0)	0.114
Stroke	4 (6.8)	8 (5.8)	0.753	10 (16.7)	0.094
Spontaneous abortion	8 (13.6)	16 (11.5)	0.686	14 (23.3)	0.170
Livedo reticularis	3 (5.1)	12 (8.6)	0.560	4 (6.7)	1.000
Thrombocytopenia	20 (33.9)	60 (43.2)	0.224	31 (51.7)	0.050

Values are presented as number (%) and mean ± SD, *p*-values were calculated by Pearson’s chi-squared test and Student’s *t*-test for two samples. Bold indicates statistically significant (*p* < 0.05) result. Abbreviations: *n*, number of patients; AB, antibody; APA: antiphospholipid antibodies, APS, antiphospholipid syndrome; n.c., not computable.

**Table 8:** Determination of the optimal cut-off value for aGAPSS as a predictive factor for cardiac manifestations using ROC analysis.

	AUC	*p*-Value	aGAPSS Cut-off Value *	Sens.	Spec.	Prev.	PPV **	NPV **
IHD	69.28	**0.003**	>8.5	80.95	55.27	8.1	13.76	97.05
AI	71.39	**0.002**	>8.5	83.33	55.00	7.0	12.23	97.77
MI ^#^	78.73	**<0.001**	>8.5	79.31	68.42	33.7	56.07	86.68
MP	69.42	**<0.001**	>8.5	80.43	59.43	17.8	30.04	93.34
TI ^#^	77.69	**<0.001**	>8.5	79.01	66.67	31.4	52.04	87.40
CM ^#^	79.01	**<0.001**	>9.5	87.50	67.36	6.2	15.05	98.79

Values are presented as percentages (%), except indicated otherwise: * aGAPSS cut-off value presented as point. Bold indicates statistically significant (*p* < 0.05) result. ** These values are dependent on disease prevalence. ^#^ AUC ≥ 75%. Abbreviations: ROC, receiver operating characteristic; AUC, area under the ROC curve; aGAPSS, Adjusted Global Antiphospholipid Syndrome Score; Sens., sensitivity; Spec., specificity; Prev., prevalence; PPV, positive predictive value; NPV, negative predictive value; IHD, ischemic heart disease; AI, aortic insufficiency; MI, mitral insufficiency; MP, mitral prolapse; TI, tricuspidal insufficiency; CM, cardiomyopathy.

## Data Availability

We cannot provide public access to individual data due to participant privacy stipulations in accordance with ethical guidelines. Upon reasonable request, qualifying researchers may apply to access an aggregated dataset by contacting the corresponding author.
